# An exploratory study of critical incidents within public organizations during leadership change

**DOI:** 10.12688/f1000research.142942.1

**Published:** 2024-04-23

**Authors:** Rian Pramana Suwanda, Fendy Suhariadi, Bagong Suyanto, Suparto Wijoyo

**Affiliations:** 1 Post Graduate School, Airlangga University, Surabaya, East Java, 60286, Indonesia; 2 Faculty of Social and Political Science, Airlangga University, Surabaya, East Java, 60286, Indonesia

**Keywords:** Leadership Change, Public Organization, Critical Incident Technique, Indonesia

## Abstract

**Background:**

Leadership changes within public organizations are often associated with achieving the organization’s vision. This exploratory study examines critical incidents and the anxiety experienced by the head of the department at the local government in the context of leadership change in the public organization. It explores anxiety, which has rarely been explored in connection with leadership change, especially with regard to public organizations and countries with a high-power distance culture. Thus, it comprehensively describes the sources, course, and consequences of anxiety due to leadership change.

**Methods:**

Critical incident technique (CIT) was used to conduct analysis because of its suitability as a theoretical framework for the exploratory nature of this research. Data were obtained through in-depth interviews from 26 informants who served as heads of departments in cities.

**Results:**

The findings revealed the causes, course, and consequence of the anxiety experienced in response to leadership change. Political choice, culture change, policy change, fear of loss, and unaccountable financing were identified as sources of anxiety. Anxiety manifested through negative, cognitive, and behavioral reactions. The consequences were divided into in-circle, out-circle, and ambivalence-circle participation.

**Conclusions:**

High-power distance culture causes leaders to portray hegemony with boundaries that are difficult to access as well as appear more directive to strengthen control within the organization. The integrated model presented here (causes, course, and consequences of anxiety) is expected to enrich the integrated, modern, and emotional science through a functional account of the emotional approach. Cognitive and affective reactions have a two-way relationship, wherein emotion influences cognition and cognition elicits emotion.

## Introduction

The new leader in Indonesia—elected through general elections—brings about massive changes to the government’s vision and mission, public service programs, and budgets and facilitates organizational change. This phenomenon is exacerbated by the harsh reality that approximately 70% of all change initiatives fail ( Beer & Nohria, 2001). Magazine articles and practitioner books have significantly shaped the 70% organizational-change failure narrative; however, such articles lacked a discussion of methods, epistemologies, and references in relation to organizational-change research and academia ( Hughes, 2011). Although previous studies have questioned the accuracy of this failure rate, organizations’ ability to successfully implement change remains critical for achieving success ( Gigliotti *et al*., 2019).

Previous studies have found that the primary determinants of successful change are employee involvement ( Gopinath & Becker, 2000; Rafferty *et al*., 2013; Rafferty & Restubog, 2010), support and trust from employees ( Kotter & Cohen, 2002), and individual readiness ( Holt & Vardaman, 2013). Other studies have proven that leadership is the key to successful change ( Colville & Murphy, 2006; Higgs & Rowland, 2001). Gilley *et al*. (2008) highlighted the importance of distinct leadership qualities and competencies for effective transformation and innovation and that change has a cascading impact on an organization. Leaders at all levels are frequently presented with concerns regarding leadership change, which allows them to create work environments that encourage change readiness ( Miles, 2001). Extensive literature on a leader’s role in successful change (e.g., Bass, 1997) has shown that although transactional and transformational leadership are complementary ( Bass & Riggio, 2006), the latter is often associated with change and innovation. Researchers have noted that different variations in change require distinct leadership styles ( Shvindina, 2017). Leaders, employees, and followers play an important role in the change effort as they actively interpret and negotiate this change ( Sahay, 2017). Therefore, organizational change requires a focus on increasing individual involvement through management models that are flexible enough to allow individual organizational interpretation ( Sheffield & Coleshill, 2001).

Change is expected to generate resistance ( Coghlan, 1993; Oreg, 2006). Resistance to change is frequently cited as a cause of the difficulty in implementing change initiatives and their failure ( Erwin & Garman, 2010): resistance is one of the most common problems faced by management when implementing change ( Bovey & Hede, 2001a, 2001b) and is the number one reason for the failure of change initiatives ( Bovey & Hede, 2001a, 2001b; Prochaska *et al*., 2001). Employee resistance is directly linked to the failure of many large-scale corporate reform efforts ( Reger *et al*., 1994). It can be argued that most organizational changes are managed from a technical perspective without understanding the effects of human elements on the success or failure of the change and ignoring the interests of equal human dimensions in implementing the change ( Beer *et al*., 1990; Huston, 1992). The attitudinal concept of resistance is divided into cognitive, emotional (frustration and anxiety), and behavioral stages ( Piderit, 2000). According to Lewin (1958), resistance to change begins with anxiety and discomfort. The early stages of change are associated with several difficulties, such as the emergence of discomfort, imbalance, and anxiety.

Uncertainty about a future event may disrupt the anticipatory process, which is a key component of adaptive cognitive responses, and lead to an overestimation of the possible threat and its severity ( Gu *et al*., 2020). Furthermore, leadership transitions are plagued by anxiety resulting in less intelligence about common situations when it is most required ( Gilmore & Ronchi, 1995). It drives many people to make more cautious decisions about sharing knowledge. Feelings of anxiety are an inevitable part of change, even in the context of public organizations. Therefore, leaders and organizations must focus on garnering commitment to change by designing coping mechanism for their followers ( Bordia *et al*., 2004a).

Experts have widely examined leadership in relation to change and leadership change. However, anxiety caused by leadership changes has rarely been studied. Most studies have explored the influence of leadership styles, such as Bono *et al*. (2007), who explained that the relationship between transformative leadership and the effective well-being of followers has positive (a composite of happiness, excitement, and optimism) and negative (a composite of anxiety, anger, and irritation) impacts. Few studies have examined the focus of leadership change, such as that of Orosz (1994) that assessed executive transition. The study found that the destructive metaphors used by a state’s agency director to describe the experience of executive transition justify the initial anxiety typically felt by employees during periods of leadership change. Thus, previous studies have failed to comprehensively describe the concept of anxiety related to leadership change.

For public organizations, the change brought about by a new leader selected through elections, is massive and radical. Previous studies have captured this phenomenon. For example, a new policy called “the best value” was proposed immediately after the 1997 elections of the Scottish local authorities ( Sheffield & Coleshill, 2001), which replaced mandatory competitive tendering with a regime that solely focused on quality issues and financial performance. Following the 2019 election cycle, Spain transitioned from an imperfect party system to a moderately pluralist party system, resulting in the world’s first coalition administration. In a short period, Spanish politics swung like a pendulum: from polarization, fragmentation, and a willingness to curtail core democratic rights in the name of Spanish unity to the ability to establish a durable accord ( Rodon, 2020). However, changes occurred before and after the elections. Government responsiveness increased significantly before the elections, as evidenced by the speed with which local governments handled requests ( Dipoppa & Grossman, 2020). The change in responsiveness by the government is in line with previous studies showing that the work done by the incumbent leader helps in the reelection of the ruling party ( Mishra & Attri, 2020).

In Indonesia, since the implementation of regional autonomy in 1999, the fate of community life, including education, health services, infrastructure development, and food security for a sustainable environment, has mostly depended on local governments. This constellation stretches as a phenomenon not only in Indonesia but also globally. Government quality is determined by its impact on citizens’ quality of life ( Huther & Shah, 1998). The main axis of regional autonomy in Indonesia is democracy driven by direct elections. Elections are the most important instrument for democratization: democracy is procedural and elections are considered the essence of democracy ( Schumpeter, 1976). Huntington (1993) believed that the democratic system alone does not suffice in elections. Free, fair, and competitive elections are possible only if there is freedom of opinion, assembly, and the press, and the candidates and opposition can criticize power without fear of reprisals. In 2020, Indonesia held simultaneous general elections in 270 regions, comprising nine provinces and 261 regencies/cities. It inaugurated 185 pairs of major or regent (leader) in February 2021, of which 114 officials served as incumbents while 255 officials were elected for the first time or called “The New Kid on the Block.” General elections in Indonesia have led to major changes in regional governance. The fundamental changes that always occur include changes in vision, which are followed by changes in programs, the selection of apparatus resources, and various policies related to public services, such as service tariffs, infrastructure development, and service management ( Smith, 2012). Additionally, leadership change within public organization is impacted by Indonesia’s high power distance culture. Hofstede (2022) measured the power distance dimension in Indonesia, which obtained a score of 78; Indonesia relies on hierarchies, unequal rights between power and non-power holders, inaccessible superiors, directive leaders, management control, and delegation. Power is centralized, and managers rely on the obedience of their team members.

Our study sought to comprehensively unravel the anxieties caused by leadership change. It studies the context of leadership changes in public organizations as a consequence of the Indonesian general election of 2020. The official heads of department in the regional government or the middle managers, who were in direct contact with the new leader, were interviewed and their responses were examined using the critical incident technique (CIT) method. A total of 26 informants were selected based on the following criteria: those who experienced critical incidents of anxiety associated with leadership change. The analysis was conducted in three stages to determine the order of concepts and themes from the causes, course, and consequences of anxiety in relation to leader change. Data validity was achieved through the support of expert judges who evaluated the consistency of the developed themes ( Flanagan, 1954; Gremler, 2004). Finally, member checking was conducted during the triangulation stage to confirm the study’s trustworthiness.

This study extends the application of CIT to the context of leadership change, which has been thoroughly explored in service-marketing research ( Grace, 2007; Gremler, 2004). Using CIT, this study proposes a model for anxiety associated with leadership change. The model comprises the causes, courses, and consequences of anxiety experienced by the head of the department in the local government. The findings are expected to expand the theory of leadership change, particularly by revealing the relationship between new leaders and their followers. Furthermore, a comprehensive description of anxiety based on Lewin’s three-phase theory is expected to deepen the understanding of the resistance that persists in the early stages of change. In addition, this research is expected to comprehensively describe the anxiety associated with leadership change in public organizations in countries with a high-power distance culture. Finally, the proposed anxiety model can deepen the study of anxiety in relation to social evaluation theory and modern emotion science.

## Literature review

### Role of leadership in change and leadership change

Extant literature suggests that leadership is strongly interconnected with change. The type of leadership is vital to the change process ( Hussain *et al*., 2018). The role of leadership in causing effective change has led to the notion that transformational leadership is a critical component of change even though both transactional and transformational leaders are considered complementary ( Bass & Riggio, 2006). Transformative leaders are charismatic, motivational, intelligent, and individualized in their approach, while transactional leaders utilize rewards and penalties to inspire individuals to enhance their organizational performance ( Bass, 1985). Given that the presence of leaders stimulates change, leaders must take the initiative by defining a vision ( Bass, 1985). Leadership style affects a group’s dynamics and the interaction between its members, which in turn influences the level of organizational readiness to change ( Wulandari *et al*., 2020). Leaders’ spirit, insight, knowledge, compassion, ideals, and learning skills are crucial aspects that lead people to embrace change ( Paterson & Cary, 2002). Leadership is the mediating force between a changing environment and an organization’s operating system (bureaucracy) ( Mintzberg, 1978). In addition, leaders’ behavior makes changing circumstances more effective ( Higgs & Rowland, 2005).

According to experts, different styles of leadership are required for different types of change. This study emphasizes the importance of specific leadership skills and characteristics required for successful change and innovation. Organizations are interested in examining and enhancing the leaders’ talent and ability to implement change and innovate at all levels in order to strengthen the change effectiveness skills ( Gilley *et al*., 2008). This distinct change relates to public organizations when a new leader causes a fundamental change. Leadership changes lead to organizational changes, such as change in the developmental vision, priority programs or innovation, selection of government apparatus resources, and various policy adjustments related to public services. This phenomenon alters the status quo—which represents stability—into either antithesis or synthesis, representing a change for worse or better, respectively ( van de Ven, 1995). Change can have a comprehensive impact on all aspects of a public organization, or at only a certain point, which is vital to support the organization; thus, change is either incremental or radical ( Tushman & Romanelli, 1985). In addition, change periodically affects medium- and long-term organizational capacity, but some are only shock effects at the beginning of the implementation; thus, change can be either sustainable or unsustainable ( Meyer *et al*., 1993). Change initiatives have become common in organizations and may range from large to medium- or small-scale ( Weick & Quinn, 1999).

Lewin’s Three-Phase Process was used as the change model in this study. The model is categorized into three stages: unfreezing, moving, and refreezing ( Lewin, 1958). The unfreezing of behavior and the establishment of a willingness to change are the first steps in the change process ( Burnes & Cooke, 2013). Lewin (1958) described unfreezing as a difficult re-education process. Moving, also known as locomotion, is the second step, which denotes a change in position within a field performed through action research ( Lewin, 1947). The final stage, freezing, denotes the permanence of a new situation ( Burnes, 2020). In organizational terms, freezing can necessitate changes in culture, norms, policies, and practices ( Armenakis *et al*., 1999). Cummings and Worley (2009) identified five critical leadership activities in the change process: motivating change, defining a vision, developing political support, managing transition, and maintaining momentum. In line with Lewin’s Three-Phase Process, motivating change and creating a vision demonstrate an unfreezing stage, developing political support and managing the transition demonstrate a moving stage of change, and maintaining momentum demonstrates the implementation and refreezing stages of change.

### Anxiety

Organizational change raises uncertainty, which is one of the most commonly reported psychological states ( Bordia *et al*., 2004a). Uncertainty has been characterized as an individual’s perceived inability to precisely foresee something ( Milliken, 1987) and has been linked to changes in processes and outcomes ( Bordia *et al*., 2004b; DiFonzo & Bordia, 1998). Bordia *et al*. (2004a) presented a three-factor paradigm of uncertainty experienced by individuals during change: strategic, structural, and job-related uncertainties. Strategic uncertainty refers to uncertainty regarding organization-level issues; structural uncertainty refers to uncertainty arising from changes in the organization’s inner workings; and job-related uncertainty refers to uncertainty related to job security, promotion opportunities, job role changes, and so on ( Bordia *et al*., 2004a). Uncertainty has several negative consequences on an individual’s well-being and satisfaction in an organizational context ( Bordia *et al*., 2004a). This leads to low morale and job satisfaction ( Bastien, 1987; Rosenfeld *et al*., 2004). It is positively related to psychological strain and negatively related to control ( Bordia *et al*., 2004b). Due to its effect on emotional states, uncertainty about future events is inextricably linked to anxiety and worry ( Gu *et al*., 2020). When changes occur very frequently, individuals are likely to feel fatigued by them and experience an increase in anxiety due to their unpredictability ( Rafferty & Griffin, 2006).

Barlow (2002) highlighted that according to Kierkegaard, the source of anxiety lies deep within an individual; anxiety is rooted in the fear of nonexistence, non-being, or nothingness, rather than the fear of death. Only by acknowledging and overcoming this dread of becoming nothing—only by facing the prospect of losing one’s identity—can one completely understand what it means to be human. Only through this experience can we make a clear separation between the self and other things or nonbeings. Other well-known theorists and clinicians have proposed comparable bases for assessing diffused and objectless anxiety. According to May (1979) and Barlow (2002), anxiety is the fear triggered by a danger to some value that the individual believes is vital to their life as a personality. The danger could be directed toward one’s physical life (threat of death) or mental well-being (threat of depression; loss of freedom and meaninglessness). Alternatively, the threat could be directed toward a value that one associates with their existence (patriotism, love for another person, “success,” etc.). Anxiety is a mixture of several emotions that can change over time and in different settings ( Izard, 1977).

The mechanisms that mediate anxiety in this study are based on the theory of social evaluation. The general criterion of social evaluation is defined as a mental process in which an individual assigns different values (positive, negative) to specific behavioral patterns (e.g., helping and hindering) that occur in social interactions (e.g., problem-solving), associates these behaviors with specific individuals (partnership values), and displays different behaviors (e.g., avoidance or preference) toward others based on their overall value ( Abdai & Miklósi, 2016). According to Manstead and Semin (1981), the negative quality of the actor’s subjective public image following a transgression is the source of social emotions resulting from unintended social infractions. The experimental results reveal that actors feel anxious as they believe others are treating them more negatively as a result of witnessing mistakes, although the situation does not affect the actor’s self-image. The fear of negative evaluations felt by actors defines social anxiety ( Button *et al*., 2015). Button *et al*. (2015) established that the learning biases of social evaluation associated with social anxiety are more strongly related to self-referential information. The anxiety experienced by the actor in the experiment was in line with social anxiety disorder, which is a severe psychiatric disorder characterized by persistent and overwhelming fears of one or more social or performance settings where there is potential for evaluation by others ( American Psychiatric Association, 2013). Fear of negative evaluation is a key component in cognitive-behavioral models of social anxiety and has rich empirical support ( Heimberg *et al*., 2010).

Over the past few decades, the study of anxiety has become increasingly integrative. Theories about anxiety have expanded based on behavior, biology, and cognition aspects, and have been integrated into modern emotion science ( Barlow, 2002). Most current theories regard emotions as fundamental action tendencies that aim to inspire conduct related to species survival. Preparing for, avoiding, and escaping potentially dangerous, life-threatening events, which are at the heart of fear and anxiety, are examples of these behaviors or action tendencies ( Barlow, 2002). Lang and McTeague (2009) regarded behavioral activities or broad reaction dispositions that occur in specific stimulus circumstances as the essence of emotion. These stimulus contexts establish the function and direction of the acts, which include not only stimulus information that inspires action but also relevant response propositions.

Furthermore, understanding anxiety through a functional account of the emotional approach places individuals and events in an inseparable unit, where events gain significance through individual efforts; thus, events and goals are interrelated ( Smit & Lazarus, 1990). According to emotion theory, functionalism is concerned with the link between feelings and what a person is trying to do, rather than evolutionary survival value ( Campos *et al*., 1994). Keltner and Gross (1999) explained the functional account of emotion, including why emotions exist and the systems of interrelated components and consequences. This functional approach provides a corridor for identifying the antecedents that mediate anxiety, as described in the social evaluation theory. Furthermore, the system of components is in line with the modern emotion science approach regarding the relevant response propositions that arise from the circumstances of a specific stimulus. However, this functional approach leads to anxiety.

Several studies have examined anxiety in various contexts, particularly health and psychology. However, anxiety related to leadership changes in public organizations has rarely been studied. The low level of research in this field may be due to some very sensitive issues that are influenced by bureaucratic cultures (e.g., high-power distance culture) in developing countries such as Indonesia. There is a phenomenon gap regarding how anxiety arises and is felt, as well as its consequences in the context of leadership changes in public organizations. In fact, research on a functional approach, the antecedents, system of components, and consequences of anxiety remains scant. There is a gap in our understanding of the variable components of anxiety associated with leadership change. Through this exploratory study of critical incidents, we examined anxiety felt by the heads of departments in local governments in response to leadership change. The study provides a framework for comprehending the problem to be solved (cause), system of components (course), and consequences of anxiety related to leadership change in public organizations. Thus, the following research question was examined: “What are the antecedents, reactions, and consequences of anxiety related to leadership changes in a public organization?”

## Methods

**Figure 1.**
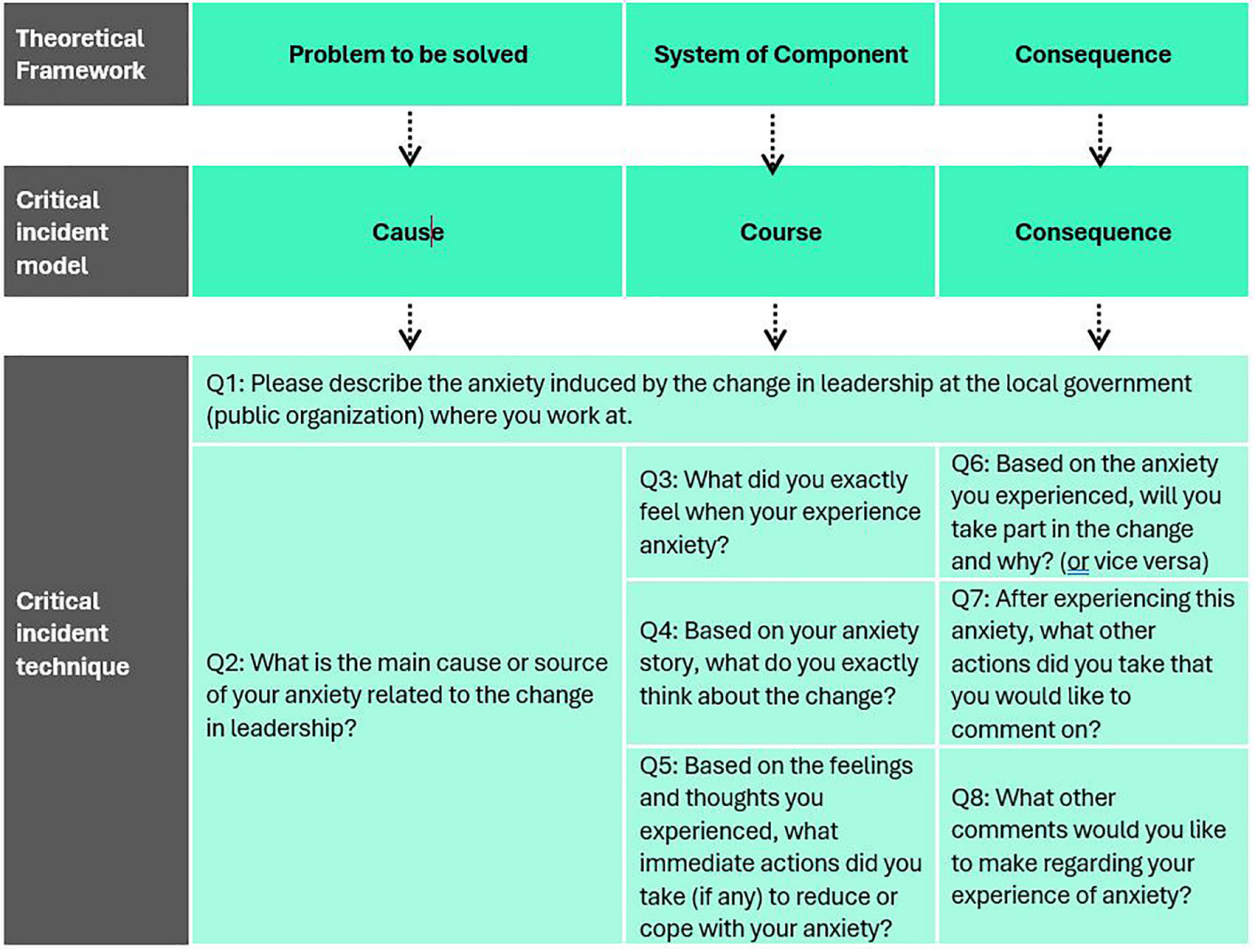
Development of the research design. This figure has been adapted from Grace, D. (2007). © 2007 by Sage Publications. Reprinted by Permission of SAGE Publication.

Figure 1 presents the research design, including the theoretical framework, models, and tactics for critical incident development (CID). We adapted the theoretical framework developed from emotional analysis and divided it into problems to be solved, system components, and consequences ( Grace, 2007). We also adopted this approach from the development of critical incident models that focus on causes, courses, and outcomes ( Edvardsson, 1992; Edvardsson & Roos, 2001). Critical incidents were mined through in-depth interviews. We used a purposive sampling method to obtain informants who uniquely followed our research interests ( Miles & Huberman, 1994; Patton, 2002).

### Critical incident technique (CIT) method

Changes caused by the new leader (regent or major elected for the first time) may be radical at any level of public organizational activity. Radical change is implemented completely if large-scale adjustments are made at the beginning of the change ( Romanelli & Tushman, 1994). In contrast, some studies have claimed that radical changes must be made gradually and carefully ( Pettigrew *et al*., 1992). However, it has been determined that early change to specific “high-impact” elements is necessary for completing radical transitions ( Amis *et al*., 2004). We believe that changes implemented either on a large scale or gradually are critical incidents that make unusual and significant contributions to human activities. This is very influential in terms of managing change, as various studies have explained that the most successful changes ensure employee involvement ( Gopinath & Becker, 2000; Holt & Vardaman, 2013; Kotter & Cohen, 2002; Rafferty *et al*., 2013; Rafferty & Restubog, 2010).

A significant contribution to human activity was found in the phenomenon of leadership changes in a public organization. It is consistent with the concept of a critical incident, which is defined as contributing significantly, either positively or negatively, to an activity or phenomenon ( Grove & Fisk, 1997). As a result, we consider the CIT to be the best tool for studying anxiety experienced by middle managers in relation to leadership change in public organizations. Furthermore, the CIT was selected based on the possible critical occurrences’ need to contextualize particular situations ( Ribeiro *et al*., 2020). In addition, CIT is an effective method for exploratory research on this little-known phenomenon ( Bitner *et al*., 1990).

The CIT is a series of processes for collecting first-hand observations of human behavior in such a way that they can solve issues and generate broad psychological principles ( Flanagan, 1954). This study adapted the theoretical model of critical incidents developed by Grace (2007) and Edvardsson (1992) to formulate the CIT method. By collecting data from the informants, we converted Grace’s (2007) self-report survey into in-depth interviews. In-depth interviews were conducted to obtain more detailed responses and help informants feel more comfortable while disclosing information about the change ( Nadia *et al*., 2020). In-depth interviews help researchers to identify the core issues of various stakeholders and prepare questions for subsequent interviews ( Malodia *et al*., 2021).

### Data collection and analytical procedures

Data were collected from June 13 to September 28, 2021, through in-depth interviews using a purposive sampling method. This flexible data mining strategy is based on the CIT, which has a flexible set of principles that must be modified and adapted to match current circumstances, as opposed to a single set of strict rules to guide data collection ( Flanagan, 1954). The inclusion criteria for informants were (1) heads of departments in the local governments of cities A, B, and C; and (2) they experienced critical incidents of anxiety associated with leadership change. The three cities held simultaneous elections on December 9, 2020, resulting in the election of a new mayor or regent for the first time. We contacted 32 candidates via social media to inform them about the research design. Additionally, we conducted telephone calls to inquire whether they had experienced critical incidents in the form of anxiety related to leadership changes. The researcher had no relationship established prior to the study’s commencement with informants from City B and C. However, we had established relationships with informants from City A in several previous studies. A total of 26 informants met the inclusion criteria and continued with in-person interviews. No one was present apart from the participants and researchers. The number of informants studied follows Flanagan (1954) that there are no strict rules regarding the appropriate sample size for CIT. We purposively selected 26 informants who had experienced anxiety in response to leadership change and uniquely fit the research interests of the study ( Miles & Huberman, 1994; Patton, 2002), and we reached data saturation.

All informants’ data were processed anonymously without distorting their scientific meaning. Twenty informants were male (76.92%), and the rest were female (23.18%). The average age was 49.8 years and the majority had entered ranks IV-A and IV-B (administrative ranks of public organization officers in Indonesia). Verbal consent was obtained from the participants to record, process, and display their statements. Consent was obtained at the opening of the interview, where the author asked for the informant’s willingness to be involved in the research. It was documented in the interview transcript. Most participants chose verbal consent due to their concerns about the research topic, which is closely related to sensitive issues and high-power distance culture in Indonesia. Written informed consent was subsequently obtained, and all participants signed the interview transcript on August, 4-10, 2023. The transcript contained additional clauses regarding informed consent, including the verbal consent obtained at the opening of the interview and the protection of participant confidentiality confirming all interview results were recorded anonymously. Ethical approval was obtained from the Postgraduate School of Airlangga University on August 10, 2023. Ethical approval was obtained retrospectively because of the urgency of our study implementation. This study was conducted after the general election which was held simultaneously in Indonesia for the first time in 2020. This first simultaneous election resulted in 63,91% of regents or mayors being elected for the first time in our research areas. The political change in the post-election period, especially within the 6 months after the election, greatly influenced informants and the culture of public organizations in Indonesia. The new regents and mayors determined a new medium-term development program and changed the bureaucratic structure, and various other fundamental changes were observed, which are thought to cause anxiety among members of public organizations. These considerations necessitated that our study began immediately to capture the critical experiences of local government officers during the leadership change. Another consideration was that our study was low risk, and the implementation and results of the study were continuously monitored by the supervisor, and supported by the continuity of the research process interactively with the informants from data collection to the preparation of the final manuscript. Our research procedures, including the consent process, were discussed in an in-depth joint review forum on July 20, 2023, with our supervisors, education staff, and lecturers from the Postgraduate School of Airlangga University. After the reviewing process, the Postgraduate School of Airlangga University stated that our study had fulfilled the ethical principles. This research complied with the principles of study ethics in the Declaration of Helsinki, as stated by the Postgraduate School of Airlangga University, Surabaya, Indonesia (Number: 2446/UN3.SPS/PT.01.06/2023).

The interview adopts a structured format with eight predetermined open questions, consistently asked to all informants. The average duration of the interviews was 47.8 minutes, and audio recordings were made. A total of 16 interviews took place in non-office settings, such as coffee shops, cafes, and restaurants, while the remaining 10 were conducted within the informant’s office. The interview transcripts were presented to the informants from October 11 to December 13, 2021, after the entire interview was completed. Participants were allowed to review and then provide approval for the interview transcripts. Furthermore, informants were offered the opportunity to review and provide input on the research results, presented in journal format, from March 1 to June 30, 2022. Additionally, before publication, verbal consent was reaffirmed in written consent with assistance from the Postgraduate School of Airlangga University from August 4 to 8, 2023.

Data saturation was achieved after the data collection and analysis. To increase the credibility of the research, we used data triangulation through multiple data sources ( Bekhet & Zauszniewski, 2012; Stavros & Westberg, 2009). Fusch *et al*. (2018) noted that triangulation involves the use of multiple external methods to collect and analyze data. Data triangulation came from two judges and three heads of local government departments from cities A, B, and C. Source triangulation enquires about the veracity of the data gathered from multiple sources. The researchers used secondary data from official websites, regional development plans, vision and mission booklets, and social media analytics to complement the interviews. The data were collected from the official websites and vision-mission booklets containing the proposed change programs endorsed by the regents and mayors. The Regional Development Plans encompass the official medium-term development plans for the municipalities during the period 2021-2024. Concurrently, social media contains a compilation of media coverage from various sources, highlighting changes in leadership in cities A, B, and C. The aim of scrutinizing this information is to comprehend the conditions of leadership change in the three cities from diverse perspectives. Consequently, the researcher expected to gain a more nuanced understanding of the information provided by the informants during interviews. For inclusion and exclusion criteria, secondary data is limited to the three city and is related to changes in leadership.

The analysis was conducted in three stages using NVivo Plus 12 for Windows. Open access alternative software besides NVivo is Taguette, ATLAS.ti, QualCoder, RQDA and MAXQDA. First, the changes perceived by the informants and the causes of anxiety associated with leadership changed were determined. Second, the course manifested by the informant in feeling, thinking, and acting immediately in response to the anxiety associated with the leadership change was examined. Third, the consequences of anxiety associated with leadership change were identified. We conducted a content analysis to improve the validity of the study, with particular attention to the concerns regarding sampling, objectivity, reliability, and systematization ( Gremler, 2004). In the second and third phases of the analysis, two judges who were experts in the fields of psychology and socio-politics were involved in the determination of the course and consequence themes.

## Results and discussion

Findings have been explained using the conceptual framework for the causes, course, and consequences of anxiety related to leadership change. Furthermore, the percentage of significant incidents discovered was used to summarize the first- and second-order concepts. Finally, we proposed an integrated model of anxiety related to leadership change in public organizations.

### Causes of anxiety

**Figure 2.**
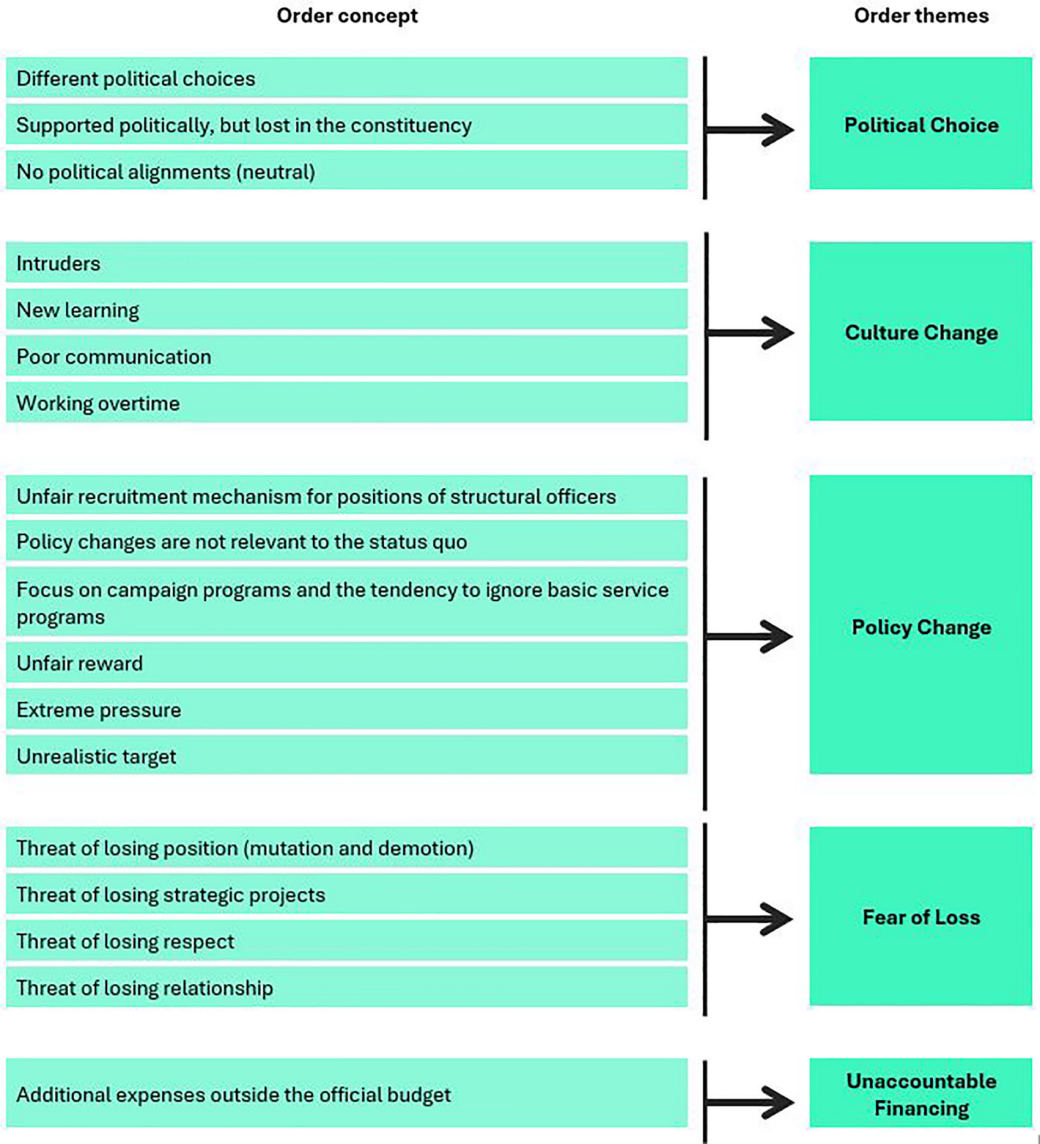
Conceptual framework for causes of anxiety associated with leader change.

The causes of anxiety related to leadership change were categorized into five themes: political choices, fear of loss, tactical budget expenditure, systems and policies, and cultural change. Figure 2 illustrates the conceptual framework for the causes of anxiety, and Table 1 summarizes the findings of critical incidents that caused anxiety (as percentages).

**Table 1.** Critical incidents causing anxiety associated with leader change.

Critical incident causing anxiety			Informant	
			Count	%
**Political choice**	(1)	Different political choices	11	42.31
	(2)	Supported politically, but lost in the constituency	8	30.77
	(3)	No political alignments (neutral)	5	19.23
			24	92.3
**Culture change**	(1)	Intruders	21	80.77
	(2)	New learning	16	61.54
	(3)	Poor communication	12	46.15
	(4)	Working overtime	6	23.08
			22	84.62
**Policy change**	(1)	Unfair recruitment mechanism for structural positions of officials	20	76.92
	(2)	Policy changes are not relevant to the status quo	16	61.54
	(3)	Focus on campaign programs and the tendency to ignore basic service programs	14	53.85
	(4)	Unfair reward	12	46.15
	(5)	Extreme pressure	8	30.77
	(6)	Unrealistic target	6	23.08
			22	84.62
**Fear of loss**	(1)	Threat of losing a position (Mutation and Demotion)	16	61.54
	(2)	Threat of losing strategic projects	9	34.62
	(3)	Threat of losing respect	8	30.77
	(4)	Threat of losing relationship	6	23.08
			20	76.92
**Unaccountable financing**	(1)	Additional expenses outside the official budget	18	69.23
			18	69.23
Total			26	100

*First theme: Political choices*

Based on in-depth interviews using the CIT, political choices are a critical cause of anxiety associated with leadership change. Of the 26 participants, 24 (92.3%) revealed that their political choices induced anxiety while facing a new leader. In-depth interviews revealed “different political choices” (42.31%) as the largest critical incident causing anxiety, followed by “supported politically, but lost in the constituency” (30.77%). Furthermore, apart from being different or supporting political choices, being neutral also caused anxiety (19.23%). The act of making a choice in a political event is always anxiety-inducing, as evidenced by the statements of several participants regarding the theme of political choice:

“So, civil servants must be neutral… we cannot turn a blind eye and we have to choose the side. The flow is like that (factual conditions observed by the informants). Now the party I choose loses like this. Yes, I am ready, fortunately, if I am still used (in the previous position of local government office), if not, I can be dumped out. Doesn’t that cause anxiety?” (Informant 1)

“It’s a secret, ya …. I was indeed a campaign team in District A (censored). But at that time, we didn’t have enough money then we lose. So, it’s clear that I’m definitely to blame!” (Informant 4)

“I didn’t choose. I am neutral. But we all know that. After this, the campaign team will surely undermine asking for a share. Those of us who are neutral will put aside. This is very unsettling. Just being professional is not enough.” (Informant 21)

Political choices put participants in a position of uncertainty, especially regarding the most widely acknowledged career paths. As stated by the participants, political choices on the losing side have a significant impact on career mutations and demotions. On the neutral side, they feel marginalized by political supporters, which causes their judgments to be sidelined. This ambiguity induces feelings of anxiety ( Tiedens & Linton, 2001; Todd *et al*., 2015). Differences in political choices relate to the inevitable diversity of opinions, values, beliefs, interpretations, and goals associated with leader change and are driven by political behavior ( Fischer *et al*., 2007; Jafariani *et al*., 2012; Petracca, 1991). In Indonesia, informants are legally prohibited from making political choices as civil servants in order to support contestants who compete in elections. However, despite this phenomenon being common, the involvement of civil servants in elections has rarely been studied academically owing to its sensitive nature. Although it is against existing norms, this political choice has a positive impact in that political behavior in organizational change is important for achieving effectiveness ( Buchanan, 2008). However, like the wheel of life, political choices that cause anxiety cause fundamental changes that determine votes ( Ladd & Lenz, 2008). Based on field findings, the informants’ various political choices were allegedly unknown to the new leader. The feelings of anxiety that arose were associated with the fear of the new leader’s view of those who did not support him or who lost the support scheme during the campaign period. This finding supports social evaluation theory, which explains that anxiety arises from the fear of negative evaluation ( Button *et al*., 2015; Heimberg *et al*., 2010; Manstead & Semin, 1981).

*Second theme: Culture change*

Culture change caused by leadership change was identified as a source of anxiety by 84.62% of the respondents. A total of 21 informants (80.77%) stated that the emergence of intruders disturbed and tended to harm the work culture of the status quo. The term intruder refers to groups outside the government bureaucracy that comprise a campaign team, such as leaders of sympathizers, party representatives, and even academics. The intruder group is considered disturbing as it fails to understand the ethics of the bureaucracy as a whole, imposes ideas, and tends to lip service. The following excerpts resonate this sentiment:

“We have to face people who claim to be the winning team. They do not understand the ins and outs of the bureaucracy, even the performance, but because they feel they are closest to the mayor, they are listened to.” (Informant 3)

“They (intruders, red) are from academics, only talk about theory, and some only tend to look for projects in local government.” (Informant 7)

“They are more lip service on our new mayor ….” (Informant 5)

In addition, the antecedents of anxiety under the cultural change theme were categorized as new learning (61.54%), poor communication (46.15%), and having to work overtime (23.08%). Critical incidents of new learning are closely related to the informants’ comfort zones. They must leave their comfortable areas and learn to support the changes launched by the new leader. At a certain level, due to policy discretion, the change was outside the scope of official duties and functions. This finding is evident from the following statements by several participants:

“It’s good like this, it works smoothly, but I was told to do this and that…the concept isn’t clear either. We have to study again.” (Informant 3)

“I’m in the field of communication and information, but I have to think about vaccine planning.” (Informant 12)

Critical incidents of poor communication relate to the communication mechanism between the informants and the new leader, either through official letters or direct interaction. Official letters, which require the leader’s decision to comply with the provisions of laws, are hampered as the leader assigns a new secretary or aide who does not have archival experience or experience with official paperwork. Direct interactions are also hampered, because the new leader’s schedule is tight. This is due to the absence of a balanced distribution system for managing official schedules. This finding is evident from the following statements by several participants:

“I have submitted a letter for the annual regional planning to get our mayor’s signature … er, it’s gone. Important papers are often lost because the mayor’s secretary is not yet skilled. The previous officer should also be used.” (Informant 8)

“Now, it’s difficult to meet the mayor because he has many events. All events, he attended. Even in one same event, he attended with the vice. This is no longer a campaign …” (Informant 17)

The critical incident of working overtime is closely related to the leader’s activities, which caused the informants to accompany the leader even outside of working hours and holidays. This finding is evident from the following statements by several participants:

“We have to be on standby for 24 hours, especially for activities related to the community, especially during a pandemic like this.” (Informant 11)

“The new mayor always wants to appear … and we have to accompany him.” (Informant 13)

Organizational culture is a valuable asset if it supports an organization’s mission, goals, and strategies. Moreover, it plays an essential role in many elements of an organization ( Calciolari *et al*., 2018; Denison & Mishra, 1995; Hartmann, 2006). The organizational culture in Indonesia, although strongly influenced by the existing political system, is unique at each level of city government. Although not studied in-depth academically, this condition is allegedly in line with Schein’s (2004) statement that organizational culture is distinctive, as it is derived from individual experience and history. This strong culture combines internal and external issues while ensuring the organization’s survival in difficult times. Furthermore, the organizational culture in Indonesia is influenced by the high-power distance culture, which is directly suspected to complicate the level of communication during situations of change. Bordia *et al*. (2004b) suggested that a systematic program of communication during change is warranted to reduce employee uncertainty.

The presence of intruders harms values and beliefs in the bureaucracy rather than new learning; having to work overtime and poor communication change habits. All these dimensions are closely related to organizational culture as a shared meaning for every member of the organization ( Robbins & Judge, 2013). These various dimensions follow organizational culture as a set of value systems, beliefs, assumptions, and norms that have been applied, agreed upon, and followed by the organization’s members for a long time as guidance for their behavior in every situation in the organization, and adapting both in internal and external environments ( Schein, 2004). Organizational culture refers to a system comprising values, beliefs, and habits that exist in the organization and interact with the formal structure, which results in the organization’s behavioral norms ( Suhariadi, 2013).

*Third theme: Policy change*

Policy change was also identified as a cause of anxiety associated with leadership change (reported by 84.62%). Here, the most crucial critical incident was the unfair recruitment mechanism for the position of structural officers (76.92%), followed by policy changes that are irrelevant to the status quo (61.54%), extending focus on campaign programs and the tendency to ignore basic service programs (53.85%), giving unfair rewards (46.15%), putting extreme pressure (30.77%), and demanding unrealistic targets (23.08%). The unfair recruitment mechanism for structural officers was stated by the informants based on their past experiences, which highlighted that every change in power, especially for those newly elected, would result in massive mutations. This mutation tends to ignore professionalism but is seen as a reward for the campaign team that supported the new leader during the campaign period. This finding is evident from the following statements made by several respondents regarding the theme of systems and policies:

“Massive mutations will occur in August (six months after the inauguration). This has been seen from the officials appointed to fill the vacant positions, right? The people appointed are the campaign team.” (Informant 25)

“As long as there is no clear blueprint, the selection of structural positions must come from the campaign team without considering credibility. For example, a sports teacher became a sub-district head, eventually went to prison…” (Informant 3)

“We struggled to start a career from the beginning. Then, just because they were close to the mayor, functional officials moved to structural without a clear assessment.” (Informant 11)

“The priority of the program is centered on populist programs during the campaign even though there are still many administrative matters and even basic services that should not be abandoned. In addition, the program target is too far away and very difficult to achieve in the next 5 years.” (Informant 12)

“We do not get the reward for the hard work we have given. Our previous achievements are unseen, only political interests …” (Informant 3)

“There is a lot of pressure from the campaign team on us to realize the change program. Is it for their interest or just delusional? But what is certain is that the budget for this year has already been determined.” (Informant 14)

“Many policies are not following the conditions of our city, especially with the detailed planning and budgeting system as it is today. Do we have to make up?” (Informant 1)

The policy change dimension relates to the implementation of organizational change policies by new leaders. This implementation is important for managing the change process to achieve developmental visions, as proclaimed by the new leader. Policy analysis has long noted the importance of clear and specific policy objectives and coherent thinking about the relationship between the initiatives to be implemented and desired outcomes ( Grizzle & Pettijohn, 2002; McFarlane, 1995).

Borrowing from Pierson’s (2000) terminology, a transformation in the policy change dimension can be called a critical juncture. The critical juncture in organizational change, especially in systems and policy changes, is a sudden impetus for change in stable institutional arrangements ( Pierson, 2000). According to various informants, the mutation and promotions for public organization officers that had been carried out for years based on the list of ranks and job evaluations were abruptly altered with mass transfers that were influenced by political interests. Thus, politics plays an important role in the overall process of policy change and reform. Politics influences the origin, formulation, and implementation of public policy, especially in the face of significant changes ( Reich, 1995). Political events increase employee anxiety because they are often volatile. This causes public organization officers to become emotionally detached and the quality of their work, in turn, declines ( Bailey & Raelin, 2015).

*Fourth theme: Fear of loss*

Fear of loss was identified as another cause of anxiety. The fear of losing a position due to mutation or demotion (61.54%), followed by the threat of losing strategic projects (34.62%), the threat of losing respect (30.77%), and the threat of losing relationships (23.08%) were gleaned from the respondents’ accounts:

“I am afraid that I will be transferred even though I have served in this office for a long time.” (Informant 1)

“… later if we move it to a ‘dry place,’ we can no longer find additional income.” (Informant 3)

“Since becoming echelon IV, I have worked in this office. It’s been a dozen years. It is difficult if you have to separate from your coffee friends (best friend).” (Informant 25).

“What if we are demoted later? Not only our income has decreased but also we have to lost respect, right?” (Informant 3)

Fear of loss is closely related to the threat of losing a position due to mutations or demotion, especially for those who serve in an influential department or have ambitions to become regional secretaries (top bureaucratic positions in local governments). Various studies have shown that loss is a source of resistance to change, such as loss of control ( Bordia *et al*., 2004b; Eby *et al*., 2000), status ( Canning & Found, 2015; Rider & Negro, 2015), comfort and privileges ( Msweli-Mbanga & Potwana, 2006), and resources ( Dubois *et al*., 2013; Shin *et al*., 2012). This dimension also resonates with Chreim’s (2006) statement that employees do not really resist change but reject perceived threats to their sense of autonomy, integrity, ideals, loss of status, salary, and comfort. Subconscious awareness of loss causes uncertainty and anxiety, which illustrates how organizations are experienced as fundamentally emotional places ( Vince, 2006).

*Fifth theme: Unaccountable financing*

Finally, 69.23% of the respondents cited unaccountable financing as a cause of anxiety associated with leadership change. This includes additional expenses that must be incurred personally or the involvement of officials in financing change programs. The following excerpts highlight this theme:

“We have to pay a fee to buy a sarong. It was used for Eid celebration.” (Informant 9)

“This year’s budget has been set, but the new mayor wants his campaign program to run. So what about the budget shortfall? Do we have to spend our own money? Yes.” (Informant 11)

The informants recounted that the new leaders proposed various new programs posed as innovations, which were outside the usual circumstances. These new initiatives were not planned or budgeted in advance, which is contrary to Indonesia’s planning and budgeting system completed one year ago. This phenomenon has caused the implementation of new programs to lead to unaccountable expenditures outside the officially available budget. The informants and their staff had to spend private money to support the program’s financing. They continued to carry out the program, although they lost money and understood that the activity was wrong in terms of planning regulations. However, they could not blame anyone, including the new leader, due to the high-power distance culture. No clear external actor can be held accountable for an increase in anxiety ( Lerner & Keltner, 2000; Wagner, 2014). Politically, an economic crisis causes anxiety if responsibility is spread and difficult to determine ( Wagner, 2014).

### Course of anxiety

**Figure 3.**
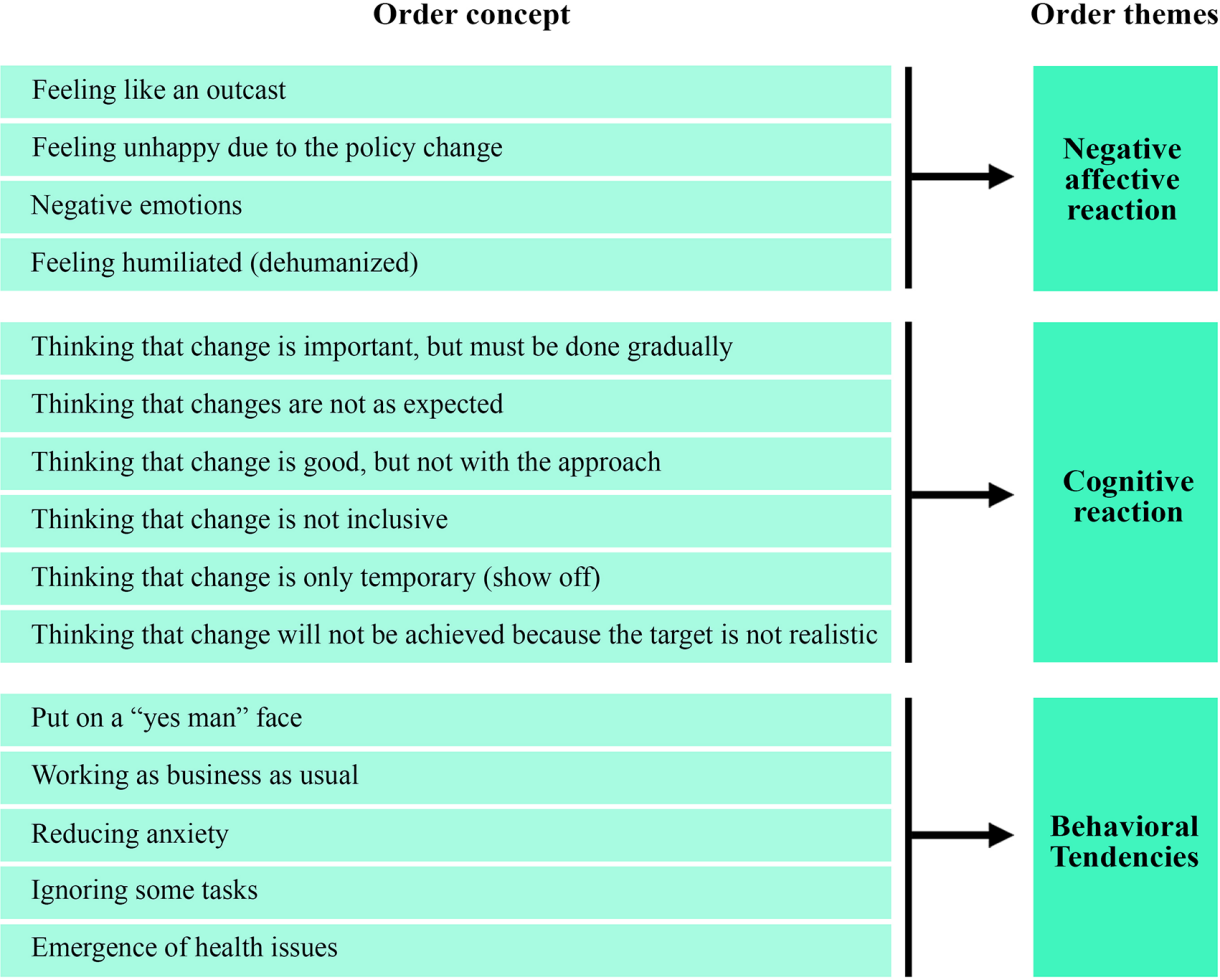
Conceptual framework for course of anxiety associated with leader change.

The course of anxiety related to leadership change was categorized into three themes: negative affective reactions, cognitive reactions, and behavioral tendencies. The conceptual framework is illustrated in Figure 3, and Table 2 summarizes the findings of critical incidents associated with the course of anxiety (as percentages).

**Table 2.** Critical incidents in the course of anxiety associated with leader change.

Critical incident in the course of anxiety			Informant	
			Count	%
**Negative affective reaction**	(1)	Feeling like an outcast	18	69.23
	(2)	Feeling unhappy due to the policy change	14	53.85
	(3)	Negative emotions	13	50.00
	(4)	Feeling humiliated (dehumanized)	6	23.08
**Cognitive reaction**	(1)	Thinking that change is important, but must be done gradually	21	80.77
	(2)	Thinking that changes are not as expected	18	69.23
	(3)	Thinking that change is good, but not with the approach	16	61.54
	(4)	Thinking that change is not inclusive	15	57.69
	(5)	Thinking that change is only temporary (show off)	12	46.15
	(6)	Thinking that change will not be achieved because the target is not realistic	6	23.08
**Behavioral tendencies**	(1)	Put on a “yes man” face	19	73.08
	(2)	Working as business as usual	16	61.54
	(3)	Reducing anxiety	12	46.15
	(4)	Ignoring some tasks	6	23.08
	(5)	Emergence of health issues	5	19.23
Total			26	100

Negative affective reactions are triggered involuntarily by emotions that lead to an individual response due to anxiety. Feeling like an outcast (reported by 69.23%) was identified as the most cited critical incident under this theme, followed by feeling unhappy due to the policy change (53.85%), negative emotions (50%), and feeling humiliated (dehumanized) (23.08%). The following statements highlight this finding:

“I felt like an outcast because I wasn’t involved in making decisions anymore. All decisions come from above (topdown) and the mayor listens more to intruders …” (Informant 3)

“Our innovations to support the change program are unseen. We have worked for decades and have idealism to serve, but we are considered as old colonial people and cannot develop.” (Informant 14)

Kantor (1923) defined affective reactions as the actual ways in which people react to affective stimuli in manners that are strikingly comparable to emotional, desire, and pain reactions. Affective reactions elicit emotions that can be differentiated based on the specificity of the original stimulus or target object ( Pieters *et al*., 1988). Kantor (1923) categorized affective stimuli into objects, events, conditions, or actions. Various studies have explained that the most proximal cause of affective reactions is events, as explained in affective events theory, which is widely recognized as the seminal explanation of the function of affection that influences employee attitudes and behaviors in the workplace ( Weiss & Beal, 2005). The results of Paterson and Cary’s (2002) study on organizational downsizing events, which are strongly associated with anxiety, support this theory. This study also demonstrates the relationship between events that cause affective reactions by establishing relevant judgments and perceptions of organizational downsizing as precursors to anxiety. Organizational downsizing as a form of change is in line with Weiss and Cropanzano’s (1996) statement that change is a potentially affective event; therefore, it is critical to analyze the emotional aspect. Paterson and Cary’s results (2002) were also supported by our findings by considering leader change as an event that triggers various affective reactions to anxiety.

Furthermore, Efendic (2017) showed that a single affective reaction or a combination of several affective reactions (negative and positive) affects decision-making. Experiments by Weiss *et al*. (1999) demonstrated positive affective reactions, such as joy, when responding to appropriate variations in procedures and distributive justice, and negative affective reactions, such as anger, that arise from biased procedures and distributive justice. Considering change as an affective event, incremental and radical changes result in positive and negative emotions, respectively ( Gersick, 1994). In our study, negative affective reactions predominated because of anxiety caused by leadership change. The study found that negative affective reactions often manifest as sadness, anger, and frustration ( Brundin *et al*., 2021; Luby *et al*., 2009; Pihkala, 2022). This finding supports Kiefer’s (2002) finding that changes in complexity are more likely to elicit negative emotions. This is also in line with previous findings that the majority of qualitative and quantitative studies on the effects of change in followers’ emotions indicate that the emotions elicited by change are primarily negative, such as anger, anxiety, and impatience ( Bartunek, 1984).

The second theme is that of cognitive reactions that were triggered by the participants’ views about change after experiencing anxiety. Cognitive reactions were manifested as a result of thinking that change is important, but it must be implemented gradually (80.77%); thinking that changes are not as expected (69.23%); thinking that change is good but not with the approach (61.54%); thinking that change is not inclusive (57.69%); thinking that change is only temporary (show off) (46.15%); and thinking that change will not be attainable, as the target is unrealistic (23.08%). This finding is evident from the following statements:

“This government is not magic, changes cannot be implemented directly “sakdet saknyet” (Javanese proverb: desire that must be achieved right away). I think this should be done gradually.” (Informant 3)

“Our government is based on Webberian principles. Many hierarchical structures must be addressed so that radical changes cannot be implemented. The changes brought now seem to only show that the campaign promises have begun to be worked on. But how about in the long term? It has not touched on the main business process, right?” (Informant 17)

“Program A (the abbreviation refers to one of mayor’s programs) is a local program. Must be aligned with regional and national programs. We must not stand alone, we must involve the parties inclusively instead of just listening to the intruder group.” (Informant 22)

Cognition is a thought process in which a person first becomes aware of stimuli, evaluates their relevance, and then explores various behavioral responses ( Scherer, 1999). Individual beliefs regarding change, whether negative or positive, are referred to as cognitive reactions ( Piderit, 2000). Cognitive and affective reactions have a bidirectional relationship in that emotion influences cognition and cognition elicits emotion ( Smit and Lazarus, 1990). In the context of change, it elicits cognitive responses (positive, negative, neutral, or mixed), which are mediated by judgments of the fairness, scale, velocity, and timing of change ( Smollan, 2006; Smollan *et al*., 2010). In our study, cognitive reactions were found with mixed evaluation criteria, where the thought of change was positive but was followed by other evaluations such as the use of wrong approach and expectations for gradual change. Piderit (2000) explains this difference regarding ambivalence, both within and among the three dimensions of attitudes toward change, namely emotional, cognitive, and behavioral, as an example of open support. This is followed by covert rejection and thoughts of accepting change, which in turn is followed by emotional resistance. Furthermore, our study found that cognitive reactions to anxiety were dominated by negative evaluations such as non-inclusive, unrealistic, temporary, and unexpected targets. These findings support previous studies on the impact of anxiety on cognitive function, such as causing problems in social and work environments ( Robinson *et al*., 2013). Moreover, Kiefer (2005) explained that employees with strong cognitive reactions are accompanied by strong negative emotions such as fear or anger and are more likely to reject change. However, Piderit (2000) explained that negative cognitive and affective responses are often well-intentioned and lead to an organizational implementation that may be more acceptable and beneficial.

The last theme is that of behavioral tendencies which refers to a controlled tendency or intention to act owing to the stimulation of anxiety associated with change. Putting on a “yes man” face (reported by 72.08%) was identified as the most cited critical incident under this theme, followed by working as business as usual (61.54%), reducing anxiety (46.15%), ignoring some tasks (23.08%), and the emergence of health issues (19.23%). The following accounts highlight this finding:

“I’ve been working for years. I have felt various changes in the four mayoral times. Well, we work as best we can, he says A we follow A, he says B we follow B.” (Informant 9)

“Bureaucracy will never change, we work systemically. Whatever the vision and mission, the programs and activities still follow the existing nomenclature.” (Informant 1)

“We don’t have to force ourselves, it’s been a few weeks since my stomach acid has recurred. Never mind, I better focus on maintaining my health.” (Informant 14)

Behavioral tendencies toward change are formed because of cognitive and emotional responses that may include both positive and negative features ( Piderit, 2000). Furthermore, Smollan (2006) explained that cognitive, affective, and behavioral responses to change are influenced by factors within the individual, change manager, and organization. As mentioned previously, the “yes man” face was the most cited behavioral response. This term describes the behavior of accepting and following change with mostly different emotional and cognitive conditions. These findings support the explanation of the ambivalence of cognitive, affective, and behavioral responses related to change ( Piderit, 2000; van Harreveld *et al*., 2015), which is open support followed by emotional and cognitive resistance. This finding is suspected to be closely related to Indonesia’s high-power distance culture. Behavioral tendencies associated with the “yes man” face are closely related to the phenomenon of unequal rights between power holders and non-power holders, but of course, more in-depth research is required to uncover them.

Another interesting finding that is influenced by high-power-distance culture is working as business as usual. This is considered a neutral behavioral tendency related to changes. The findings revealed that two middle managers preferred to carry out routines as usual or avoid conflict. The high-power distance and bureaucratic organizational cultures in Indonesia encourage middle managers to choose their behaviors more deeply. This consideration mechanism was expressed by Piderit (2000), who considered the implications of behavioral choices before a behavior was chosen. Additionally, ignorance of some tasks can be considered a form of resistance to leadership change, which is in line with Oreg’s (2006) study. These three forms of behavioral tendencies are behavioral intentions in the form of a tendency to support or reject change ( Cunningham *et al*., 2002; Madsen *et al*., 2005; Martin *et al*., 2005).

Reducing anxiety and the emergence of health issues are considered as a form of neutral reaction related to change from an organizational viewpoint. Such behavior tends to affect middle managers’ personalities, both mentally and physiologically. This finding supports Wisse and Sleebos’s (2016) finding that change is a major stress factor for employees and is mediated by feelings of uncertainty. Furthermore, organizational change has a short-term effect on mental health decline ( Bamberger *et al*., 2012). These findings also support Greenglass and Burke’s (2000) finding that organizational change is correlated with depression and anxiety and is a crucial point for organizational members to manage.

### Consequence of anxiety

**Figure 4.**
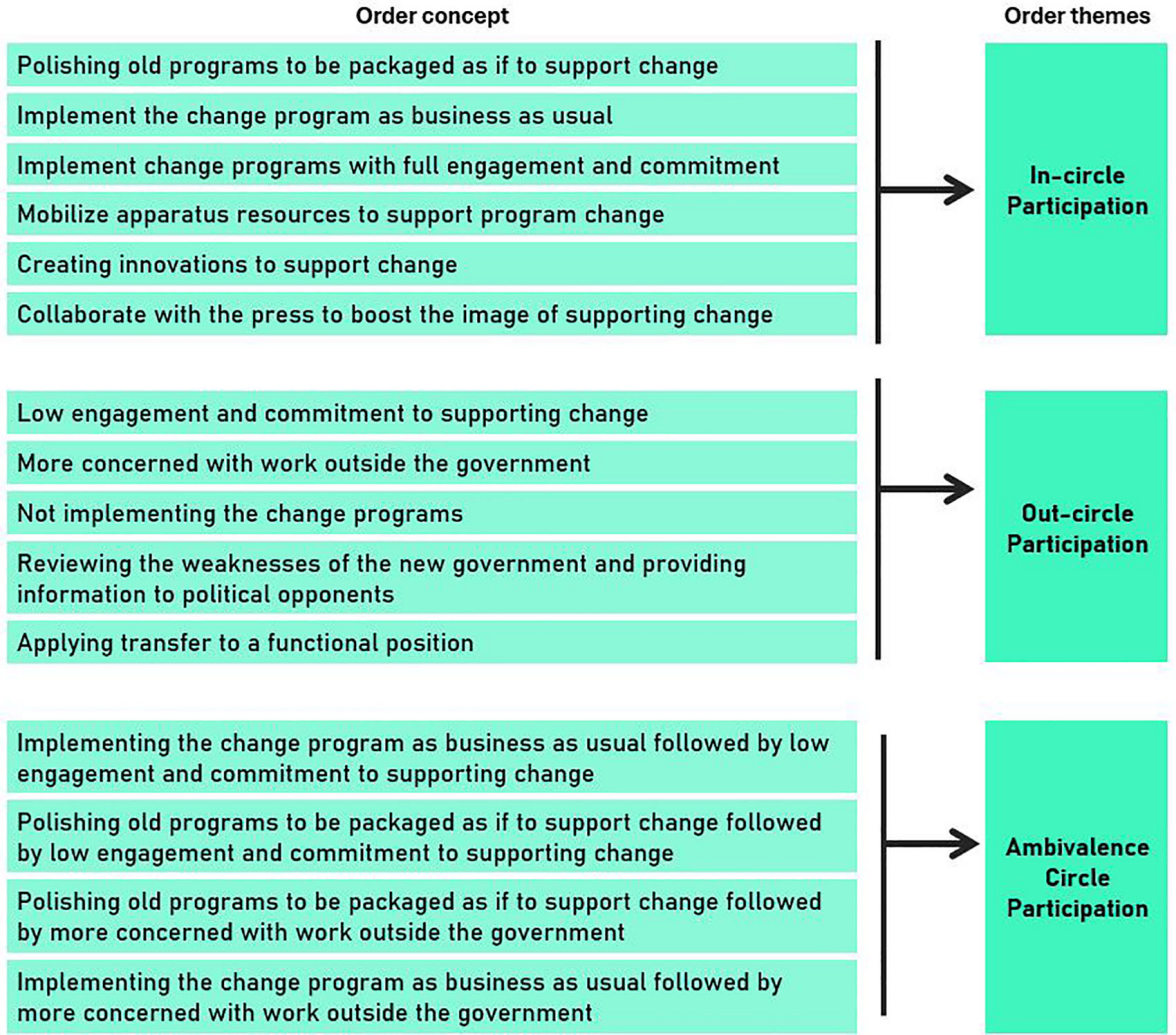
Conceptual framework for consequences of anxiety associated with leader change.

The consequences of anxiety associated with leadership change were categorized into three sequential themes: in-circle, out-circle, and ambivalent circle participation. “Circle” relates to Indonesian culture, which denotes people close to power as “inside the circle” and vice versa. The conceptual framework related to this section is illustrated in Figure 4, and Table 3 summarizes the findings of critical incidents under the consequence of anxiety (as a percentage).

**Table 3.** Critical incidents in the consequences of anxiety associated with leader change.

Critical incident in the consequences of anxiety			Informant	
			Count	%
**In-circle participation**	(1)	Polishing old programs to be packaged as if to support change	15	57.69
	(2)	Implementing the change program as business as usual	11	42.31
	(3)	Implementing the change programs with high engagement	6	23.08
	(4)	Mobilizing apparatus resources to support program change	6	23.08
	(5)	Creating innovations to support change	5	19.23
	(6)	Collaborating with the press to boost the image of supporting change	4	15.38
**Out-circle participation**	(1)	Low engagement to support the change	11	42.31
	(2)	More concern with work outside the government	6	23.08
	(3)	Not implementing the change programs	3	11.54
	(4)	Reviewing the weaknesses of the new government and providing information to political opponents	3	11.54
	(5)	Applying transfer to a functional position	2	7.69
**Ambivalence circle participation**	(1)	Implementing the change program as business as usual followed by low engagement and commitment to supporting change	6	23.08
	(2)	Polishing old programs to be packaged as if to support change followed by low engagement and commitment to supporting change	6	23.08
	(3)	Polishing old programs to be packaged as if to support change followed by more concerned with work outside the government	3	11.54
	(4)	Implementing the change program as business as usual followed by more concerned with work outside the government	3	11.54
Total			26	100

“In-circle participation” is a category in favor of the leader change. The critical incident related to this category was polishing old programs to be packaged as if they supported change (57.69%). This was followed by implementing the change programs as business as usual (42.31%), implementing the program changes with high engagement (23.08%), mobilizing apparatus resources to support the program change (23.08%), creating innovations to support the change (19.23%), and collaborating with the press to improve the image of supporting the change (15.38 %). The following statements highlight these findings:

“However, we must support this change, but we must be smart to take advantage of the situation. Not all new programs are innovations. From my various experiences over the years, we have to carry out rebranding programs, old programs in line with the changes brought by the new mayor.” (Informant 6)

“Vision and mission may change, but the program and activities remain the same. Many old programs are good and useful and can still be maintained. Many previous programs are almost similar to new programs that’s why we try to polish this program so that it can support the change.” (Informant 14)

“Out-circle participation” refers to the resistance against the leadership change. The critical incidents associated with this theme were low engagement to support the change (42.31%), more concern with work outside the government (23.08%), not implementing change programs (11.54 %), reviewing the weaknesses of the new government, providing information to political opponents (11.54%), and applying transfer to a functional position (7.69%). The following statements highlight these findings:

“I’ve been lazy to hear the rhetoric of change, let it happen.” (Informant 2)

“We see the new mayor doesn’t think of us as an outcast, I’d better focus on working on my property business.” (Informant 15)

“This year’s budget has been determined. We will not implement these change programs.” (Informant 7)

“Let me work as usual. We’ll see how this new government goes. Forced changes will never move the wheels of the bureaucracy. I’m ready to be the opposition.” (Informant 3)

“This change is extremely stressful. Work is no longer comfortable because it is full of risks. I’ve decided to move to functional.” (Informant 1)

“Ambivalent circle participation” refers to the participation that supports change, but also disguises resistance. The critical incidents associated with this theme were implementing the change program as business as usual followed by low engagement and commitment to supporting change (23.08%), polishing old programs to be packaged as if to support change followed by low engagement and commitment to supporting change (23.08%), polishing old programs to be packaged as if to support change followed by more concern with work outside the government (11.54%), and implementing the change program as business as usual followed by more concern with work outside the government (11.54%). The following statements highlight these findings:

“Let’s just use the old program, we adapt it to support it, but that’s how it is, I’ll just let it go along. We are tired of too much branding.” (Informant 10)

“What do we do, we’ve just run the program as usual. I can still walk while looking for money outside.” (Informant 23)

The results reveal that 50% of the informants showed support for the leadership change or were categorized as in-circle participants, 23.08% of the informants who indicated resistance were categorized as out-circle participants, and 26.92% of the informants were included in the category of ambivalent circle participation.

Bovey and Hede (2001a) found that behaviors in response to change included supportive versus opposing, active versus passive, and covert versus overt behaviors. Based on this, “in-circle participation” tends to be supportive, active, and open. In this study, the consequences of anxiety that support leadership change manifest as explicit behavior through active involvement in change ( Cunningham *et al*., 2002). The most dominant and interesting consequence of this category is the polishing of old programs to be packaged as if to support change. Although it seems tricky, this phenomenon can be considered as the creativity of a middle manager who traditionally describes the key levels of analysis as individual, group, and organizational, with creativity culminating at higher levels as a result of creative output at lower levels ( Woodman *et al*., 1993). Further studies are required to examine the level of creativity as an adaptive process of change. However, Ford (1996) explained that a low level of engagement can also produce creativity, which may not be novel or useful, while high engagement can encourage a person’s ability to produce creative outcomes. Polishing creations can be considered an improvisation of using problem-focused coping strategies and adjusting to change ( Amiot *et al*., 2006) as a self-efficacy to execute the courses of action required to manage prospective situations ( Bandura, 1977; van Dam *et al*., 2008).

We also found that collaboration with the press to improve the image of middle managers is a marker of supporting change. From the perspective of change, the communication process is vital for realizing effective change as an inherent process of change implementation, including the dissemination of the value of change, training, feedback, and interaction ( Lewis & Seibold, 1998). The main goal of communication is to reduce employee uncertainty ( Bordia *et al*., 2004b). In this case, the informant used the communication system not only to disseminate the value of change but also to imagine themselves as an actor who supports change.

The extreme resistance observed in this study was highlighted by the informants’ decision to switch to a functional position, meaning leaving their position as the head of a local department in the local government (structural position) and moving to a functional position, which is based on function and expertise and is more independent (e.g., Widyaswara or lecturer). This implies that the informant left the organization. The study found that informants who chose this extreme measure felt strong negative emotions, such as the feeling of dehumanization. This finding supports Kiefer’s (2005) study, which claimed that negative emotions indicate an essential organizational judgment and a behavioral tendency to withdraw both in the short- and long-term. Kiefer (2005) claimed that employees who experience strong cognitive reactions and strong negative emotions are more inclined to reject change. This finding also supports Oreg’s (2006) study, which explains that someone who experiences anxiety, anger, and stress due to change has a greater intention to leave the organization.

Other manifestations of extreme resistance besides withdrawal included disloyalty, neglect ( Turnley & Feldman, 1999), and sabotage ( Spector & Fox, 2002). We found disloyal resistance and neglect in the decision to not implement the program that was concerned with work outside the organization. This study found that middle managers preferred to run organizational programs that have been planned and budgeted in advance. The tight planning and budgeting regulations in Indonesia with information system-based supervision were cited as reasons for not changing program implementation in the current fiscal year, despite the new leader initiating a change program. We also found disloyalty and neglect associated with working outside public organizations, such as being a consultant or running a business. Finally, resistance in the form of sabotage manifested in the form of finding new government mistakes and providing this information to political opponents. Furthermore, Gaubatz and Ensminger (2017) stated that resisting change can be a “contention” that continues to disrupt and frustrate change efforts.

These four forms of out-circle participation can be categorized as forms of irrational resistance, which refers to the term used by de Jager (2001) as resistance to change for its own sake. Rational resistance is referred to as low involvement in implementing changes or being passive ( de Jager, 2001). In this study, we found low involvement in the form of loss of focus, low involvement in task coordination, decreased productivity, and decreased enthusiasm for supporting change. Here, “engagement” refers to a positive, fulfilling, work-related state of mind that is characterized by vitality, dedication, and immersion ( Schaufeli & Bakker, 2004) and is categorized as job satisfaction, job involvement, organizational commitment, and empowerment ( Macey & Schneider, 2008). Low engagement in the short- and long-term will cause leadership changes to fail, considering that various previous studies have explained that the main determinant of successful change requires employee involvement ( Gopinath & Becker, 2000; Rafferty *et al*., 2013; Rafferty & Restubog, 2010). Furthermore, Neubert and Cady (2001) considered employee engagement in change programs and investigated its association with crucial organizational outcomes ( Bordia *et al*., 2004b).

Finally, ambivalent circle participation was also a form of consequence. This circle denotes behavior that supports change but with covert resistance. This theme signifies ambivalence within and among the three dimensions of attitudes to change, namely emotional, cognitive, and behavioral, as an example of open support followed by covert rejection ( Piderit, 2000). Previous studies have shown that an ambivalent approach affects physical behavior ( Schneider *et al*., 2015). van Harreveld *et al*. (2015) described the consequences of ambivalence on influence, cognition, and behavior, and how these different categories relate to each other and to their source, where affection plays the most central role.

## Conclusions

**Figure 5.**
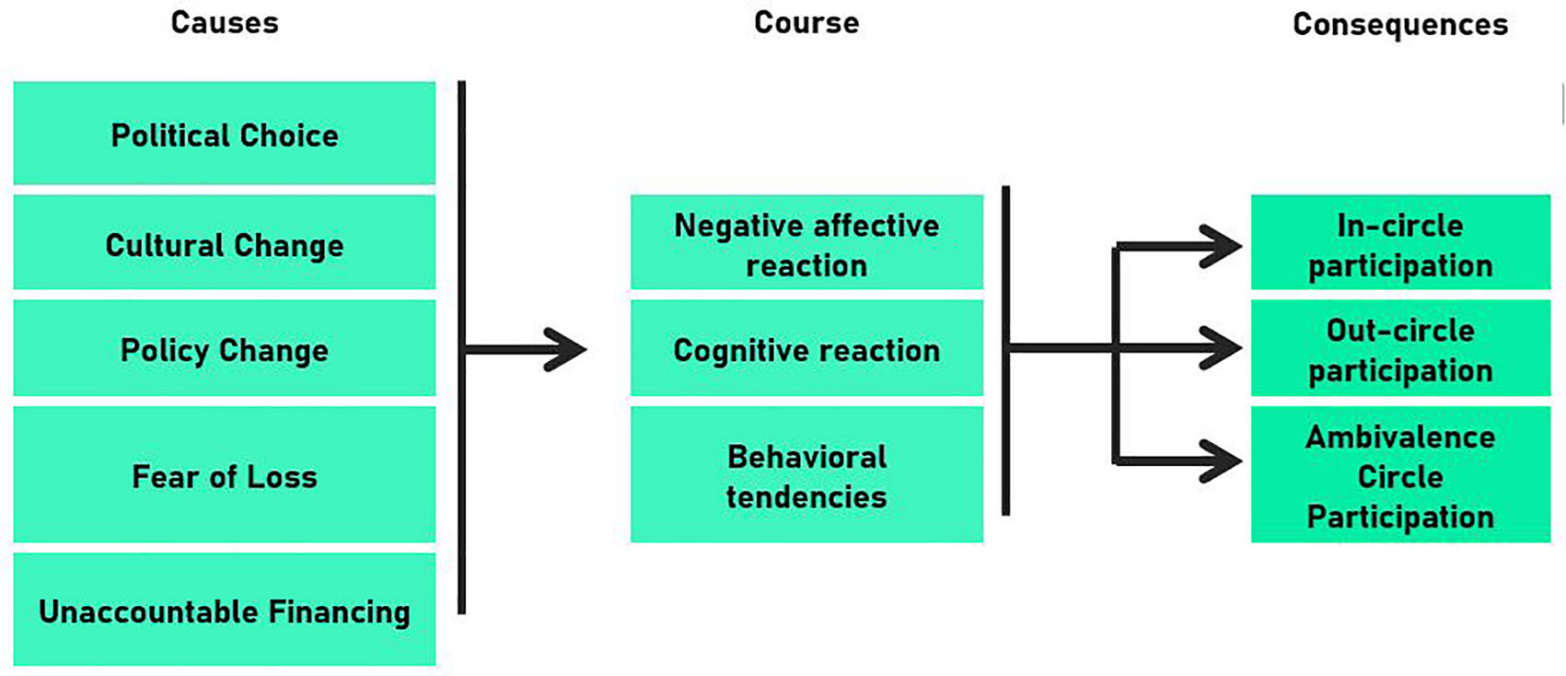
Integrated model of anxiety associated with leader change.

Our study contributes to the systematic formulation of an integrated model of the causes, courses, and consequences of anxiety associated with leadership change ( Figure 5). This study identifies critical incidents that explain the phenomenon of anxiety, which has rarely been investigated in relation to leadership change, especially in public organizations.

First, our study extends extant literature on the important role of leader-follower relationships in creating successful change ( Colville & Murphy, 2006; Gopinath & Becker, 2000; Higgs & Rowland, 2001; Holt & Vardaman, 2013; Kotter & Cohen, 2002; Rafferty *et al*., 2013; Rafferty & Restubog, 2010). Scholars have extensively researched the relationship between leadership and change and leadership change. However, this study deepens our understanding and comprehensively describes the concept of anxiety associated with leadership change. The crucial factor that increases the difficulty of research is sensitivity related to political power and organizational culture, especially in the context of Indonesia’s high-power distance culture. Thus, the organizational culture in Indonesian bureaucracy is closely related to the hierarchy that creates inequality between power and non-power holders ( Hofstede Insights, 2022). Leaders display hegemony with boundaries that are difficult to access, are more directive, and try to strengthen control within the organization.

Second, this research provides empirical evidence for Lewin’s (1947) Three-Phase Process, wherein difficulties, such as discomfort, imbalance, and anxiety, are experienced in the early stages of change. Our findings also support those of Gilmore and Ronchi (1995): leadership transitions are plagued with anxiety. The consequences of this finding reinforce the concept of successful change management, not only from a technical point of view, but also from the human dimension ( Beer *et al*., 1990; Huston, 1992).

Third, the integrated model of anxiety associated with leadership change is expected to enrich understanding of social anxiety in social evaluation theory and integrated modern emotion science. The findings of this study support social evaluation theory in that they put fear of negative evaluation as the key component that causes anxiety ( Button *et al*., 2015; Heimberg *et al*., 2010; Manstead & Semin, 1981). Integrated modern emotion science is enriched through a functional account of the emotion approach. Cognitive and affective reactions have a two-way relationship in which emotion influences cognition and cognition elicits emotion ( Smit & Lazarus, 1990; Smollan, 2006). Furthermore, ambivalence exists within and among the three dimensions of attitudes toward change, namely emotional, cognitive, and behavioral ( Piderit, 2000; van Harreveld *et al*., 2015). We also found open support followed by covert rejection and thoughts of accepting change followed by emotional resistance (negative affection). Finally, our study advances the application of critical incident techniques in the context of leader change, especially in public organizations, which has previously been explored in service marketing research ( Grace, 2007; Gremler, 2004).

Practically, this study makes a valuable contribution for practitioners in managing the anxiety that arises in response to leadership change. First, understanding the causes, course, and consequences of anxiety in the context of leadership change allows practitioners to choose a management strategy that places humans on an equal dimension in the change process. Understanding the human dimension is important because the key component of successful organizational change is the involvement of human resources, leaders, and followers ( Gopinath & Becker, 2000; Holt & Vardaman, 2013; Kotter & Cohen, 2002; Rafferty *et al*., 2013; Rafferty & Restubog, 2010). Second, an understanding of anxiety induced by leadership change, especially in public organizations, can be a key consideration in bureaucratic talent management policies. This knowledge can be used by the government or regulator to develop a blueprint for the apparatus of public organizations in a meritocratic manner. This consideration is crucial in the face of Indonesia’s high-power distance characteristics, which directly affect the culture of public organizations. Thus, understanding the key role of human resources in change is expected to induce major improvements for the government in strengthening the professionalism of public organizations.

However, this study has several limitations. First, leadership change in public organizations is a sensitive issue that makes bureaucrats (public organization officers) averse to discussing it, especially in Indonesia. This limitation needs to be investigated further in different contexts, such as developed countries with high-power distance cultures and types of informants that include members of the organization, considering that all informants in our study are middle managers. Second, future studies can develop empirical frameworks, such as compiling measurement items for anxiety related to change; examining the relationship between causes, course, and consequences of anxiety; and formulating a prescriptive strategy to reduce anxiety associated with leadership change.

## Data Availability

Figshare: Underlying data for ‘Exploratory study of critical incidents within public organization leadership change’, https://doi.org/10.6084/m9.figshare.25282879.v1 ( Suwanda, *et al.*, 2024). This project contains the following underlying data;
(1) Interview guidelines and written consent; (2) Recapitulation of Interview Transcripts; and (3) Coding of interview results. Data are available under the terms of the Creative Commons Attribution 4.0 International license (CC-BY 4.0).
