# Supplementary material for: An exploratory study of critical incidents within public organizations during leadership change
Source: F1000Res. 2026 Feb 28;13:357. Originally published 2024 Apr 23. [Version 2] doi: 10.12688/f1000research.142942.2 (PMC12989057; doi:10.12688/f1000research.142942.2)
Supplement: Peer review history

# Reviewer report: Reviewer response for version 2

**DOI:** 10.5256/f1000research.196888.r463113

**Published:** 2026-03-14

**Authors:** Siti Kholifah

**Affiliations:** 1 Universitas Brawijaya, Malang, East Java, Indonesia

I have carefully reviewed the version of the manuscript in light of the three concerns I raised in my previous report. I am pleased to confirm that all three points have been adequately addressed:

 1. Theoretical integration: the revised manuscript now explicitly connects the results and discussion sections., including  affective events theory, cognitive appraisal and ambivalence theory and the functional perspective of emotion. The bidirectional relationship between affective and cognitive reactions is also clearly articulated.

 2. Depth of analysis: the analysis of political choices as a source of anxiety has been substantially expanded. The authors now explain how different political alignments (supporting the winning candidate, the losing candidate, or remaining neutral) generate distinct patterns of uncertainty and behavioral responses, supported by illustrative informant excerpts. 

 3. Practical implications: this section has been significantly strengthened with concrete, actionable recommendations for policymakers, including structured leadership transition programs, merit-based recruitment safeguards, inclusive communication strategies adapted to high power-distance contexts, and institutional psychological support systems. 

 In view of these revisions, I am satisfied that the authors have responded constructively and thoroughly to my previous comments. I am therefore pleased to amend my status from Approved with Reservations to Approved.

Is the work clearly and accurately presented and does it cite the current literature?

Partly

If applicable, is the statistical analysis and its interpretation appropriate?

Partly

Are all the source data underlying the results available to ensure full reproducibility?

Yes

Is the study design appropriate and is the work technically sound?

Yes

Are the conclusions drawn adequately supported by the results?

Yes

Are sufficient details of methods and analysis provided to allow replication by others?

Yes

Reviewer Expertise:

My research areas in sociology of politic, sociology of religion and gender studies

I confirm that I have read this submission and believe that I have an appropriate level of expertise to confirm that it is of an acceptable scientific standard.

# Reviewer report: Reviewer response for version 1

**DOI:** 10.5256/f1000research.156546.r282573

**Published:** 2025-09-11

**Authors:** Sabar Sabar

**Affiliations:** 1 Institut Teknologi Sepuluh Nopember, Surabaya, Indonesia

If applicable, is the statistical analysis and its interpretation appropriate?

1. No measures of intercoder reliability are reported — while common in qualitative work, this would strengthen claims about consistency.
2. At times, percentages are presented with high precision (e.g., 92.3%), which may give a false sense of “quantitative exactness” given the small N=26. Rounding (e.g., 92%) could look more natural.

Are all the source data underlying the results available to ensure full reproducibility?

1. Raw interview transcripts: These are not included or made available as supplementary data. Without them, external researchers cannot fully verify coding or theme development.
2. Coding framework: The manuscript describes stages of analysis but does not publish the codebook or detailed coding tree used to move from raw transcripts → first-order concepts → second-order themes.
3. Data management: It is unclear whether transcripts were analyzed manually or with software (e.g., NVivo, Atlas.ti).
4. Access to secondary sources: While official documents are cited, no dataset or repository is provided linking the specific materials used.
5. Confidentiality: Given the sensitivity of the informants (public officials, political context), full raw data sharing may not be ethically possible.

Is the work clearly and accurately presented and does it cite the current literature?

Yes

If applicable, is the statistical analysis and its interpretation appropriate?

Partly

Are all the source data underlying the results available to ensure full reproducibility?

Partly

Is the study design appropriate and is the work technically sound?

Yes

Are the conclusions drawn adequately supported by the results?

Yes

Are sufficient details of methods and analysis provided to allow replication by others?

Yes

Reviewer Expertise:

Strategic Management and change

I confirm that I have read this submission and believe that I have an appropriate level of expertise to confirm that it is of an acceptable scientific standard.

# Response

**Published:** 2026-02-19

**Authors:** Rian Pramana Suwanda

**Affiliations:** Airlangga University, Indonesia

**If applicable, is the statistical analysis and its interpretation appropriate?**

- **No measures of intercoder reliability are reported — while common in qualitative work, this would strengthen claims about consistency.**

Thank you for this insightful comment. We agree that reporting measures of intercoder reliability can strengthen confidence in the consistency of qualitative findings.

 This study employed a qualitative Critical Incident Technique (CIT) approach using NVivo Plus 12, with a structured multi-stage coding process. The initial coding was conducted systematically, followed by iterative comparison across cases. In the second and third phases of analysis, two independent judges with expertise in psychology and socio-politics were involved in reviewing and validating the categorization of themes related to the course and consequences of anxiety. Discrepancies were discussed until interpretive agreement was reached.

 While statistical indices such as Cohen’s kappa are more commonly associated with positivist content analysis, our study prioritized analytical rigor through expert validation, peer debriefing, systematic coding procedures, and transparent documentation of theme development. We have clarified this process more explicitly in the revised manuscript to enhance transparency and demonstrate consistency in the analytical procedure.

- **At times, percentages are presented with high precision (e.g., 92.3%), which may give a false sense of “quantitative exactness” given the small N=26. Rounding (e.g., 92%) could look more natural.**

We appreciate the reviewer’s comment regarding numerical precision. Given the relatively small sample size (N = 26), reporting percentages with decimal precision (e.g., 92.3%) may indeed give an impression of excessive quantitative exactness.

 In the revised manuscript, percentages have been rounded to whole numbers where appropriate to improve clarity and avoid overstating precision. We confirm that this adjustment does not affect the substantive interpretation of the findings.

**Are all the source data underlying the results available to ensure full reproducibility?**

**Thank you for the important question regarding data availability.**

  

- **Raw interview transcripts: These are not included or made available as supplementary data. Without them, external researchers cannot fully verify coding or theme development.**

Thank you for highlighting the importance of full verification. We would like to clarify that the anonymized raw interview transcripts, along with the structured coding outputs, have been made publicly available in the repository at:

https://doi.org/10.6084/m9.figshare.25282879.v1

 The shared materials allow external researchers to trace the progression from raw transcripts to coded segments and identified themes. By examining the anonymized transcripts alongside the documented coding structure, independent researchers can assess how first-order concepts were derived and how they were aggregated into higher-order themes.

 We hope this ensures sufficient transparency for verification of the coding process and theme development while maintaining ethical safeguards.

1. **Coding framework: The manuscript describes stages of analysis but does not publish the codebook or detailed coding tree used to move from raw transcripts → first-order concepts → second-order themes.**

Thank you for this constructive suggestion. The detailed coding framework, including the progression from raw transcripts to first-order concepts and second-order themes, is available in the public repository at:

https://doi.org/10.6084/m9.figshare.25282879.v1

 The repository includes the structured coding outputs that document how themes were developed from the interview data. We have also clarified this in the revised manuscript to enhance transparency and reproducibility of the analytic process.

  

- **Data management: It is unclear whether transcripts were analyzed manually or with software (e.g., NVivo, Atlas.ti).**

Thank you for the request for clarification. As stated in the manuscript, the analysis was conducted in three stages using **NVivo Plus 12 for Windows**. The software was used to organize, code, and categorize the interview transcripts systematically.

 The analysis involved:

 (1) identifying perceived changes and causes of anxiety,

 (2) examining the course of anxiety in terms of feelings, thoughts, and actions, and

 (3) identifying the consequences of anxiety associated with leadership change.

 To strengthen validity and reliability, content analysis procedures were applied, and in the second and third phases two independent judges with expertise in psychology and socio-politics were involved in validating the course and consequence themes.

 We have clarified this more explicitly in the revised manuscript to enhance methodological transparency.

- **Access to secondary sources: While official documents are cited, no dataset or repository is provided linking the specific materials used.**

Thank you for this important point. The secondary sources cited in the manuscript (e.g., official regulations, policy documents, and public government reports) are publicly accessible documents issued by authorized institutions and were used for contextual reference rather than as a primary dataset for analysis.

 Because these materials are publicly available and formally published by government bodies, they were cited directly in the reference list rather than deposited in a separate repository. The empirical findings of this study are based on interview data, which are available in anonymized form in the public repository at:

https://doi.org/10.6084/m9.figshare.25282879.v1

 We have clarified this distinction in the revised manuscript to improve transparency.

- **Confidentiality: Given the sensitivity of the informants (public officials, political context), full raw data sharing may not be ethically possible.**

Thank you for highlighting the ethical considerations. We fully agree that confidentiality is critical given the political sensitivity of the informants (public officials in a leadership transition context).

 To address this, we have made the raw interview transcripts publicly available in anonymized form at:

https://doi.org/10.6084/m9.figshare.25282879.v1

 All identifying information, including names, institutional identifiers, cities, and politically sensitive references, has been carefully redacted to prevent deductive disclosure. This approach ensures transparency and reproducibility while maintaining strict adherence to ethical standards and participant confidentiality.

# Reviewer report: Reviewer response for version 1

**DOI:** 10.5256/f1000research.156546.r282574

**Published:** 2024-09-05

**Authors:** Siti Kholifah

**Affiliations:** 1 Universitas Brawijaya, Malang, East Java, Indonesia

I have some comments regarding this article:

 1. The study uses some theories to frame the findings. However, the integration of these theories into the analysis could be more explicit. For instance, when discussing the cognitive and affective reactions, it would be helpful to directly tie these reactions back to specific theoretical concepts, illustrating how the data supports or challenges existing models. This is important to strengthen the theoretical contribution of the research.

 2. This study provides a broad categorization of the causes, course and consequences of anxiety to the leadership change. The categorization is useful, but there is aneed for deeper analysis. For instance, the findings on political choices as a source of anxiety could benefit from further explanation of how different political alignments specifically influence anxiety levels and behaviour among department heads, also can provide an example. 

 3. The study explains valuable insights into the anxiety experienced by public organization leaders during leadership changes. However, the practical implication of these findings could be expanded. Consider providing more concrete recommendations for policymakers on how to address and manage the anxieties identified. This could include suggestions for leadership training, communication strategies, or organizational policies that could help mitigate the negative effect of anxiety and improve the overall success of leadership transitions in high-power distance cultures like Indonesia.

Is the work clearly and accurately presented and does it cite the current literature?

Partly

If applicable, is the statistical analysis and its interpretation appropriate?

Partly

Are all the source data underlying the results available to ensure full reproducibility?

Yes

Is the study design appropriate and is the work technically sound?

Yes

Are the conclusions drawn adequately supported by the results?

Yes

Are sufficient details of methods and analysis provided to allow replication by others?

Yes

Reviewer Expertise:

My research areas in sociology of politic, sociology of religion and gender studies

I confirm that I have read this submission and believe that I have an appropriate level of expertise to confirm that it is of an acceptable scientific standard, however I have significant reservations, as outlined above.

# Response

**Published:** 2026-02-19

**Authors:** Rian Pramana Suwanda

**Affiliations:** Airlangga University, Indonesia

**First Review**

***The study uses some theories to frame the findings. However, the integration of these theories into the analysis could be more explicit. For instance, when discussing the cognitive and affective reactions, it would be helpful to directly tie these reactions back to specific theoretical concepts, illustrating how the data supports or challenges existing models. This is important to strengthen the theoretical contribution of the research.***

 We sincerely thank the reviewer for this insightful comment. We agree that clearer theoretical integration strengthens the contribution of the study. In response, we have substantially revised the Results and Discussion sections to make the linkage between empirical findings and the theoretical framework more explicit and systematic.

 Specifically, we have strengthened the integration of theory in the following ways:

- First, in the section on negative affective reactions, we now explicitly connect leadership change as an organizational event to Affective Events Theory (Weiss & Cropanzano, 1996), clarifying how leader transition functions as a salient affective stimulus that triggers emotions such as exclusion, humiliation, and frustration. We also clarify how these emotional responses are intensified in high power-distance contexts.
- Second, in the section on cognitive reactions, we explicitly relate participants’ evaluative judgments to theoretical concepts of cognitive appraisal and ambivalence in change (Piderit, 2000; Smollan, 2006). We clarify how anxiety mediates evaluative processes concerning fairness, velocity, inclusiveness, and feasibility of change. The bidirectional relationship between affective and cognitive reactions (Smit & Lazarus, 1990) is now clearly articulated.
- Third, in the section on behavioral tendencies, we explicitly frame compliance, withdrawal, routine maintenance, and covert resistance as functional and strategic responses to anxiety, grounded in the functional perspective of emotion (Barlow, 2002) and ambivalence theory (Piderit, 2000). This clarifies how affective and cognitive reactions translate into observable behavioral positioning.
- Finally, in the consequences of anxiety section, we more clearly conceptualize “in-circle,” “out-circle,” and “ambivalent circle participation” as relational positioning outcomes shaped by anxiety within high power-distance bureaucratic structures. This extends existing models of change response by incorporating hierarchical proximity to power as a culturally embedded consequence of anxiety.

Through these revisions, we aim to demonstrate more clearly how the empirical data not only illustrate existing theoretical models (e.g., affective events theory, uncertainty theory, ambivalence toward change) but also extend them by situating anxiety within leadership transition processes in high power-distance public organizations.

 We believe these revisions significantly enhance the theoretical clarity and contribution of the manuscript.

**Second Review**

**This study provides a broad categorization of the causes, course and consequences of anxiety to the leadership change. The categorization is useful, but there is aneed for deeper analysis. For instance, the findings on political choices as a source of anxiety could benefit from further explanation of how different political alignments specifically influence anxiety levels and behaviour among department heads, also can provide an example.**

 We thank the reviewer for this valuable suggestion. We agree that deeper analytical elaboration strengthens the explanatory power of the categorization presented in the study.

 In the revised manuscript, we have expanded the analysis of political choices as a source of anxiety by clarifying how different forms of political alignment (supporting the winning candidate, supporting the losing candidate, and remaining neutral) generate distinct patterns of uncertainty and behavioral responses among department heads.

 Specifically, we now explain that:

- Department heads who supported the losing candidate experienced heightened job-related and structural uncertainty, particularly regarding potential demotion, mutation, or marginalization.
- Those who remained politically neutral reported anticipatory anxiety stemming from fear of exclusion from informal networks of influence (“intruders”) despite formal neutrality.
- Those aligned with the winning camp demonstrated more adaptive and strategic positioning behaviors (e.g., rebranding old programs to align with new leadership narratives), which correspond to in-circle participation.

We have also incorporated illustrative excerpts from informants to demonstrate how political alignment translated into differentiated anxiety levels and behavioral tendencies. This clarification makes explicit the mechanism through which political positioning influences both the intensity and manifestation of anxiety.

 Thus, while the original categorization was maintained, the revised manuscript provides a more nuanced explanation of how political alignment shapes uncertainty, emotional reactions, and subsequent behavioral positioning within a high power-distance bureaucratic context.

**The study explains valuable insights into the anxiety experienced by public organization leaders during leadership changes. However, the practical implication of these findings could be expanded. Consider providing more concrete recommendations for policymakers on how to address and manage the anxieties identified. This could include suggestions for leadership training, communication strategies, or organizational policies that could help mitigate the negative effect of anxiety and improve the overall success of leadership transitions in high-power distance cultures like Indonesia.**

 We sincerely thank the reviewer for this constructive suggestion. We agree that expanding the practical implications strengthens the contribution of the study, particularly given the applied nature of leadership transitions in public organizations.

 In the revised manuscript, we have substantially expanded the Practical Implications section to provide more concrete and actionable recommendations for policymakers and practitioners.

 Specifically, we now:

- Propose structured leadership transition programs, including early communication forums, participatory vision-alignment workshops, and transparent clarification of short-, medium-, and long-term policy roadmaps to reduce strategic and job-related uncertainty.
- Recommend strengthening merit-based recruitment and promotion safeguards during leadership transitions to minimize politically driven mutations and enhance perceived fairness.
- Suggest inclusive communication strategies tailored to high power-distance contexts, including structured feedback channels and cross-level consultation mechanisms to reduce covert resistance and “yes-man” compliance behaviors.
- Recommend leadership development programs focusing on emotional intelligence, change communication competence, and participatory governance.
- Highlight the importance of institutional psychological support systems (e.g., stress management programs, well-being monitoring, confidential counseling mechanisms) to mitigate prolonged anxiety effects.

These additions explicitly connect the empirical findings on the causes, course, and consequences of anxiety with practical governance reforms, particularly in high power-distance bureaucratic environments such as Indonesia.

 We believe that these revisions meaningfully strengthen the applied relevance of the study and address the reviewer’s concern **.**
